# Based on network pharmacology and in vitro experiments to prove the effective inhibition of myocardial fibrosis by Buyang Huanwu decoction

**DOI:** 10.1080/21655979.2022.2084253

**Published:** 2022-06-21

**Authors:** Tianyue Wang, Xinyu Jiang, Yanmin Ruan, Jun Zhuang, Yuanjun Yin

**Affiliations:** a The 2nd Clinical Medical College, Zhejiang Chinese Medical University, Hangzhou, China; b The 1st Clinical Medical College, Zhejiang Chinese Medical University, Hangzhou, China; c Department of Physiology, Zhejiang Chinese Medical University, Hangzhou, China

**Keywords:** Myocardial fibrosis, Buyang Huanwu decoction, network pharmacology, molecular docking, IL-17 signaling pathway

## Abstract

Among cardiovascular diseases, myocardial fibrosis (MF) is a major pathological change underlying heart failure and is associated with a high mortality rate. However, the molecular mechanism underlying MF has remained elusive. Buyang Huanwu decoction (BYHWD), a traditional Chinese medicine (TCM) formula for cardiovascular diseases, exhibits good anti-inflammatory and blood-activating properties. In the present study, we studied the MF inhibitory effect of BYHWD using network pharmacology and experimental validation. We used several databases to collect information on MF and related drugs and finally obtained cross-targets for BYHWD and MF. After that we got protein-protein interaction (PPI) network and performed gene ontology (GO) and Kyoto encyclopedia of genes and genomes (KEGG) pathway enrichment analyses to obtain key signaling pathways for further study. After screening, interleukin (IL)-6, IL-1β, and matrix metallopeptidase 9 (MMP9) were selected for *in vitro* experiments, which included cell cycle studies, cell migration rate, quantitative reverse transcription-polymerase chain reaction (qRT-PCR), and western blotting (WB). Finally, molecular docking was performed to validate the results. We found 299 common targets between BYHWD and MF. In total, 75 core targets of the PPI core network were selected for enrichment analysis, and the IL-17 signaling pathway, which is intricately linked to inflammation, was speculated to be involved. Accordingly, *in vitro* experiments were performed. Altogether, our findings indicated that BYHWD can affect the function of cardiac fibroblasts and reduce the expression of inflammatory factors in rats. In summary, BYHWD can inhibit MF by reducing the expression of inflammatory factors and affecting the IL-17 signaling pathway.

## GRAPHICAL ABSTRACT

**Figure:**
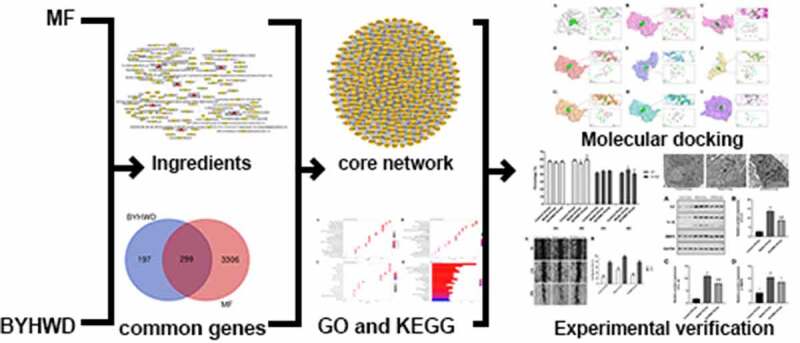

## Highlights

- BYHWD attenuates the inflammatory response in MF.
- BYHWD affects the expression of IL-6, IL-1β, and MMP9 in RCFs.
- BYHWD may affect the IL-17 signaling pathway.
- Network pharmacology revealed an association between MF and IL-17.
- Quercetin, luteolin, and paeoniflorin may be important active elements in BYHWD.

## 1. Introduction

Cardiovascular diseases are the leading cause of death worldwide and impose a substantial economic burden on society [1]. Thus, it is highly crucial to prevent and treat such diseases and conduct related research. MF is a standard pathological change occurring during the development of several cardiovascular diseases. In addition, it is a pathological hallmark of several cardiovascular diseases and participates in the development of heart failure, myocardial infarction, hypertension, and diabetes [2]. Furthermore, it causes remodeling of the heart and disrupts the balance between the production and degradation of extracellular interstitial matrix [3,4]. In general, MF causes stiffness of the myocardium, thereby affecting the systolic and diastolic functions of the left ventricle of the heart and increasing the mortality rate over time [5]. It is currently believed that the development of MF is intricately linked to the excessive proliferation of fibroblasts [6] and the accumulation of extracellular matrix in the interstitial space [7], both of which are essential pathological factors in the development of MF. In certain cardiovascular diseases, an effective strategy to prevent and treat the long-term outcome in patients with ventricular remodeling is to prevent MF [8]. Unfortunately, no effective drugs are available to control MF [4].

In China, TCM is being used as an effective complementary treatment for several diseases. TCM uses a mixture of herbs and the synergistic effects of small molecules of active ingredients of these herbs to facilitate the treatment of disease [9,10]. As a traditional formula for treating Qi deficiency and blood stasis, BYHWD has been recorded in Yi Lin Gai Cuo for hundreds of years [11]. This formula consists of seven herbs, namely, *Astragalus membranaceus* (Huang Qi), *Angelica sinensis* (Dang Gui), *Radix Paeoniae Rubra* (Chi Shao), *Lumbricus* (Di Long), *Ligusticum chuanxiong* (Chuan Xiong), *Carthamus tinctorius* (Hong Hua), and *Semen Persicae* (Tao Ren) [12]. Previous studies have demonstrated that BYHWD can benefit Qi, activate blood circulation, improve coronary circulation, and reduce myocardial ischemia [13–15].

Presently, numerous studies have focused on treating neuroinflammation induced after an ischemic stroke with BYHWD. A few experimental results have shown that this treatment can directly and effectively inhibit MF; however, the therapeutic mechanism has remained unclear.

To more systematically and comprehensively elucidate the mechanism of the drug in treating MF, we decided to use a network pharmacology approach. Network pharmacology, a popular analytical method, has been extensively used in the research of herbal compounds [16]. Network pharmacology uses the existing databases by constructing network diagrams to obtain the relationship between drugs and diseases [17]. In addition, it uses GO and KEGG enrichment analyses to better reveal the underlying molecular mechanisms and relevant signaling pathways by which drugs exert their therapeutic effects. The relevant conclusions obtained through network pharmacology can efficiently provide the basis for conducting subsequent experiments [18].

In this study, we used a network pharmacology approach to screen active ingredients of the above-mentioned seven herbs and construct a network relationship map with the disease-related differential genes as well as performed enrichment analysis and molecular docking. We hope that network pharmacology can integrate the relevant contents of biology and pharmacology to study the relationship between drug-target-disease in a comprehensive and multifaceted manner [19]. Furthermore, using cellular experiments, we demonstrated that BYHWD inhibits the development of MF and simultaneously validated the obtained results to a certain degree. We believe that this approach will allow us to decipher the relevant mechanistic details, which will provide a reference value for conducting further in-depth studies in the future.

## 2. Materials and methods

### 2.1 Network pharmacology analysis

#### 2.1.1. Collection and screening of active ingredients and targets of drugs

For the seven drugs in BYHWD, we used the traditional Chinese medicine systems pharmacology (TCMSP) database (https://tcmspw.com/tcmsp.php) to collect the active ingredients of the drugs [20]. During the screening, oral bioavailability (OB) ≥ 30% and drug-likeness (DL) ≥ 0.18 were used as the filtering criteria. For the herb *Lumbricus*, we used the Bioinformatics Analysis Tool for Molecular mechANism of Traditional Chinese Medicine (BATMAN-TCM) database (http://bionet.ncpsb.org/batman-tcm/) with a score cutoff ≥ 2.0, *P* ≤ 0.05 as the screening conditions. For functional ingredients obtained from the search, corresponding databases were used to summarize the details of relevant potential targets for further processing and analysis.

#### 2.1.2. Constructing a network diagram of the relationship between drugs and active ingredients

Cytoscape is visualization software that is used to represent network relationships by constructing network diagrams [21]. The collected active ingredients of the drug were imported into and visualized using Cytoscape_v3.8.2.

#### 2.1.3. Collection of disease targets

The GeneCards (https://www.genecards.org/) database [22] was used to obtain the disease target data using ‘myocardial fibrosis’ as the keyword.

#### 2.1.4. Acquisition of common targets for BYHWD and disease

For the targets obtained using the TCMSP database, we used the UniProt database (https://www.uniprot.org/) to convert them into uniform gene names and then imported them into the ‘Draw Venn Diagram’ online tool (http://bioinformatics.psb.ugent.be/webtools/Venn/) to produce Venn diagrams and derive the cross-targets of BYHWD and diseases.

#### 2.1.5. Construction of PPI network diagram

Previously obtained common gene data were imported into the STRING database (https://cn.string-db.org/). The STRING database [23] allows the retrieval of protein interaction relationships – expressed as a confidence score for protein interactions. All protein interaction data are weighted and integrated and have a calculated reliability value. To create PPI network diagrams, we selected the species as ‘*Homo sapiens*’ and exported the data results as a ‘tsv’ file. This file was entered into Cytoscape_v3.8.2 to generate the PPI network diagram.

#### 2.1.6. Acquisition of core network

We used the CytoHubba plugin in Cytoscape_v3.8.2 and applied the maximal clique centrality (MCC) algorithm to the obtained PPI network graph and then obtained the top 10 proteins of relative importance and ranked them. Next, the PPI network map was further refined using the Molecular Complex Detection (MCODE) plugin, from which the core network was obtained for subsequent analysis.

#### 2.1.7. GO and KEGG pathway enrichment analyses

GO and KEGG enrichment analyses were performed using the Hiplot online tool (https://hiplot.com.cn/). Hiplot, a visualization-based statistical analysis tool, is a simplified use of the R language. The R package ‘clusterProfiler’ is applied to the enrichment tool [24]. The core targets obtained through the MCODE plugin were entered into the website, and the KEGG database was selected. The enrichment minimum gene set was not less than 10, and the top 20 enrichment entries were displayed. After computational analysis, the results are presented as bar graphs or bubble charts. The GO enrichment analysis presents biological processes BP, cellular compositions CC, and molecular functions MF, whereas KEGG enrichment primarily reveals pathway-related content. The specific information of the enrichment was exported as an ‘xlsx’ file.

### 2.2. In vitro *experimental validation*

#### 2.2.1. Reagents

The following reagents were used: Dulbecco’s modified Eagle medium (DMEM) (Hangzhou Jinuo Biology, Art No. GNM-12800-s); superior fetal bovine serum (Sijiqing, Art No. 13011–8611); 0.025% trypsin (TBD, Art No. TE2004Y); CCK-8 Kit (Boster Bioengineering Co., Ltd., Wuhan, Art No. AR1160); Ang II (Sigma Aldrich, Art No. A9525-10 MG); 4’,6-diamidino-2-phenylindole (DAPI) staining solution (Solarbio, Art No. C0065); TRIzol Plus RNA Purification Kit (Invitrogen, Art No. 12183–555); RNase-free DNase set (Qiagen, Art No. 79254); SuperScript III First-Strand Synthesis SuperMix for quantitative reverse transcription-polymerase chain reaction (qRT-PCR) (Invitrogen, Art No. 11752–050); and Power SYBR Green PCR Master Mix (Applied Biosystems, Art No. 4367659); bicinchoninic acid (BCA) protein concentration determination kit (enhanced) (Beyotime, Art No. P0010); anti-IL-6 primary antibody (Abcam, Art No. Ab259341); anti-IL-1β primary antibody (Abcam, Art No. Ab254360); anti-MMP9 primary antibody (Abcam, Art No. Ab283575); anti-GAPDH primary antibody (Abcam, Art No. Ab181602); goat anti-rabbit IgG (H + L) secondary antibody (Thermo Pierce, Art No. 31210); and goat anti-mouse IgG (H + L) secondary antibody (Thermo Pierce, Art No. 31431).

#### 2.2.2. Experimental animals and cells

Three-to-four-month-old specific pathogen-free (SPF)-grade Sprague-Dawley (SD) male rats weighing 250 g were obtained from the Shanghai Shrek company. They were kept in the experimental animal center of Zhejiang Chinese Medical University. The room temperature was maintained at 22°C, relative humidity ranged from 40% to 60%, and 12 h light/12 h dark cycles were used. Animals were allowed access to food and water freely. Rat cardiac fibroblasts (RCFs) were obtained from Shanghai Qincheng (Art No. QC1388, Batch No. 20190828). All animal experimental protocols and methods were approved by the Experimental Animal Ethics Committee of Zhejiang Chinese Medical University (No.: ZSLL-2018-054).

#### 2.2.3. Cell culture

RCFs were cultured in high-glucose DMEM containing 10% fetal bovine serum in a 5% CO_2_ incubator. The cells were passaged and used for experiments to grow to approximately 80% confluence.

#### 2.2.4. Preparation of BYHWD

The composition of BYHWD was as follows: *Astragalus membranaceus* 60 g, *Angelica sinensis* 10 g, *Ligusticum chuanxiong* 10 g, *Radix Paeoniae Rubra* 10 g, *Lumbricus* 10 g, *Carthamus tinctorius* 5 g, and *Semen Persicae* 5 g, making up a total of 110 g. The restorative materials were purchased from the pharmacy of the TCM outpatient department of Zhejiang Chinese Medical University. The above seven kinds of TCM in the form of slices and totaling 110 g were added to 1100 g of distilled water and soaked for 2 h, boiled for 0.5 h on military fire, slow decoction for 1 h on civil fire, and filtered. The filtrate was collected, extracted again with 660 g of distilled water according to the above steps, combined 2 times the filtrate, and concentrated by rotary evaporator to the final raw drug mass concentration of 2 g/mL (in raw drug quantity).

#### 2.2.5. Preparation of medicated serum

Twenty-eight male SD rats were equally divided into a control group and an experimental group. The experimental group received 1 mL/(kg. D) BYHWD by oral gavage, whereas the control group was administered the same amount of normal saline twice a day for 3 days. Blood was collected by cardiac puncture 2 h after the last gavage. Animals were anesthetized with 10% chloral hydrate, placed in the supine position, and a 2-cm needle was inserted under the xiphoid process to collect blood from the heart. Blood was placed in 15-mL centrifuge tubes and left at room temperature for 2 h; the serum was separated by centrifugation at 4000 rpm for 12 min. The upper clear serum layer was collected. The serum samples from the control rats and BYHWD-treated rats were heat-inactivated at 56°C for 30 min, sterilized by passing through 0.22-μM filter membranes, aliquoted into 2-mL cryopreservation tubes, and stored at −80°C for future use.

#### 2.2.6. Effect on cell migration

RCFs were seeded into a 6-well plate at a concentration of 1 × 10^5^ cells per well. Cells were incubated with 10% fetal bovine serum for 12 h and subjected to serum-free synchronization for 12 h. After changing the culture medium, the cells were divided into control, model, and BYHWD groups, where DMEM without serum was added to the control group and DMEM containing 10^−7^ mol/L ANG II was added to the model and BYHWD groups. After 24 h of treatment, three scratches were made in each well with 200-μL pipet tips, and cells were washed thrice with phosphate-buffered saline (PBS). Afterward, 10% control rat serum was added to the control and the model groups, and 10% serum from the BYHWD-treated rats was added to the BYHWD group. At 0 h, 24 h, and 48 h after dosing, the scratches were observed and imaged using a camera attached to an inverted phase-contrast microscope. ImageJ software was used to evaluate the scratched areas and calculate the cell mobility. Cell mobility% = (0 h scratch area–scratch area after culture)/0 h scratch area.

#### 2.2.7. Effect on cell cycle

The cells were seeded and treated as described above. The cells were collected at 24 h and 48 h after treatment and were rinsed with PBS thrice. Next, 0.25% trypsin was added to each well for cell digestion for 2 min, and 10% fetal bovine serum was added to inactivate trypsin. Cells were collected, centrifuged at 1000 rpm for 5 min, the liquid was discarded, cells were washed with PBS twice, fixed in 70% pre-cooled ethanol, and stored at −4°C until analysis. For analysis, cells were first centrifuged at 2000 rpm for 5 min, ethanol was discarded, cells were washed with PBS twice, DAPI dye solution was added, and the cells were stained in the dark for 20 min. After centrifugation, cells were washed with PBS twice and suspended; next, 20 μL of the content of each well was taken in 2-mL EP tubes, and the data were collected by flow cytometry. The Klaus software (Beckman company) was used for analyses and data processing.

#### 2.2.8. Observation of cellular ultrastructure

The cells were seeded and treated as described above. The cells were treated with drugs and after 48 h, the cells were washed twice with PBS, incubated with 1 mL/well of 2.5% glutaraldehyde for 2 h, scraped, collected, centrifuged, and then washed twice with PBS. After centrifugation, fresh glutaraldehyde was added to disperse the cells for fixation. Subsequently, the cells were rinsed with phosphoric acid, fixed with 1% osmic acid, rinsed with phosphoric acid, dehydrated in a step-wise manner with increasing concentrations of ethanol, embedded in pure acetone and embedding solution, and solidified at different temperatures. Afterward, the cells were sliced with an ultrathin microtome, double stained with 3% uranyl acetate and lead citrate, and observed under a transmission electron microscope.

#### 2.2.9. Determination of relative mRNA levels of IL-6, IL-1β, and MMP9

The cells were seeded and treated as described above. After 48 h, the 6-well plate was washed with PBS twice, and 1 mL of the TRIzol reagent was added to extract the total RNA of RCFs. After determining its content, purity, and quality by ultraviolet (UV) spectrophotometry and electrophoresis, the RNA was kept at −80°C until use. For reverse transcription, RNA was used as a template to synthesize cDNA. The reaction volume was 20 μL, and the reaction conditions were 25°C for 10 min, 50°C for 30 min, and 85°C for 5 min. For real-time PCR, Primer Premier 6.0 and Beacon Designer 7.8 software were used for quantitative PCR primer designing (Shanghai Sangon Bioengineering Co., Ltd.). The primer sequences are listed in Table 1. Glyceraldehyde 3-phosphate dehydrogenase (GAPDH) was used as an internal reference gene, and *IL-6, IL-1β*, and *MMP9* genes were amplified. The reaction conditions for the real-time PCR amplification system were 95°C, 1 min; 40 cycles of (95°C, 15s; 63°C, 25s; collecting fluorescence). After amplification, the PCR products were slowly heated from 55°C to 95°C to establish a dissolution curve. Each sample was analyzed thrice, and the relative expression of each gene was established using the following formula: 2 ^(Ct of internal reference gene-^^Ct^ ^of target gene)^.

**Table 1.** Primers for qRT-PCR

Gene name		Primer Sequences
IL6	Forward Reverse	CTTCACAGAGGATACCACCCACA
		CAGTGCATCATCGCTGTTCATACA
IL-1β	Forward Reverse	CCTAGGAAACAGCAATGGTCGGGAC
		GTCAGAGGCAGGGAGGGAAACAC
MMP9	Forward Reverse	GCAAACCCTGCGTATTTCCAT
		GATAACCATCCGAGCGACCTTT
GAPDH	Forward Reverse	GAAGGTCGGTGTGAACGGATTTG
		CATGTAGACCATGTAGTTGAGGTCA

#### 2.2.10. WB

The cells were seeded and treated as described above. After 48 h of treatment with the drug, the total proteins were prepared using the whole protein extraction kit and quantified using the BCA quantitation kit. Finally, 60 µg of protein was separated by sodium dodecyl sulfate–polyacrylamide gel electrophoresis (SDS-PAGE) electrophoresis. The separated proteins were transferred to a membrane under 100 V and constant pressure for 2 h. The membrane was blocked in a sealed bag with 5% powdered skim milk at room temperature for 1 h before overnight incubation with primary antibodies (1:10000 for GAPDH, 1:1000 for IL-6 and MMP9, and 1:500 for IL-1β) at 4°C. The membrane was rinsed with TTBS (a mixture of tris-buffered saline [TBS] and polysorbate 20 [Tween 20]) and incubated with a secondary antibody on a shaking table at room temperature for 1 h; the membrane was washed with TTBS. One milliliter of electrochemiluminescence (ECL) working solution was prepared using SuperSignal West Dura Extended Duration substrate and the membrane was incubated in it at room temperature for 1 min. The excess ECL reagent was removed, the membrane was sealed, and an X-ray film was positioned in a cassette and exposed for 5 to 10 min. ImageJ software was used to analyze the band intensity. Each band was measured thrice. The results are expressed as the mean ± standard deviation (SD).

#### 2.2.11. Statistical analysis

The SPSS 20.0 software was used for statistical analysis. All data are expressed as mean ± SD. One-way analysis of variance (ANOVA) [25] was used to analyze the data, and the difference was considered significant when *p* < 0.05.

### 2.3. Molecular docking

After obtaining the enriched signaling pathways by KEGG enrichment analysis, we selected pathways closely related to MF for molecular docking experiments. First, genes involved in the selected signaling pathways were built into the results obtained from CytoHubba plug-in analysis. We traced back the essential genes to the corresponding active ingredients for molecular docking. The compounds of interest were searched through the PubChem database (https://pubchem.ncbi.nlm.nih.gov/) to download the structures in the SDF format, which was converted to mol2 format files using the Chem3D software. The protein structures were downloaded in the PDB format from the RCSB database (https://www.rcsb.org/). Afterward, we used the Pymol software to remove solvent molecules and ligands, followed by the AutoDock Tools 1.5.6 software to add hydrogen, calculate charges, and assign atomic types. Finally, the data were saved in the pdbqt format. Molecular docking was performed with Autodock Vina 1.1.2 [26], and the docking assemblies were finally visualized and analyzed using Discovery Studio 2020.

## 3. Results

The aim of this study was to investigate the potential mechanism of action of BYHWD against MF. First, we used a network pharmacology approach to predict the potential targets of action and related signaling pathways involved in MF using BYHWD. Next, we validated these results by conducting cellular experiments. Finally, the relevant active ingredients in BYHWD were molecularly docked with certain key proteins to demonstrate the pharmaceutical efficacy of BYHWD laterally using computer simulation of molecular interactions.

### 3.1. Establishment of a BYHWD database and visualization of the drug–active ingredient relationship network diagram

**Figure 1.**
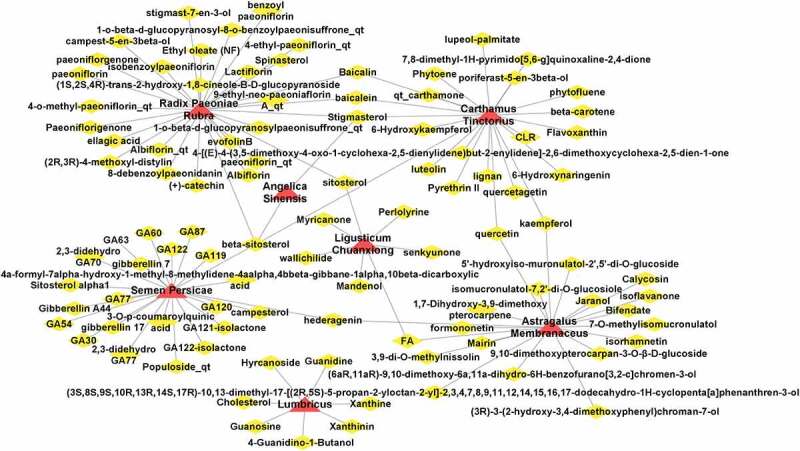
Diagram showing BYHWD herbal and active ingredients. Yellow diamond nodes represent active ingredients, whereas red triangle nodes represent herbs.

The TCMSP and BATMAN-TCM databases were used to screen 98 active ingredients associated with seven herbs and 496 potential gene targets built on the input parameters of the ADME correlation model. Figure 1 shows the corresponding results between herbs and active ingredients imported into Cytoscape_v3.8.2 for visualization, which showed that four herbs, namely, *Radix Paeoniae Rubra, Semen Persicae, Astragalus membranaceus, Carthamus tinctorius* possessed more active ingredients and potential targets.

### 3.2. Obtaining common targets of BYHWD and MF and constructing the active ingredient-common target-disease relationship map

**Figure 2.**
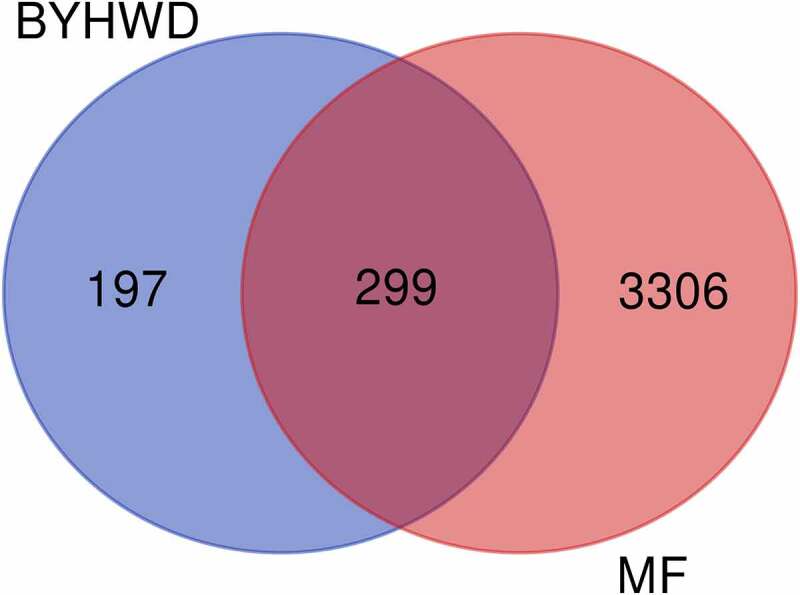
Venn diagram of common targets of BYHWD and MF. The number of common targets for both is 299.

**Figure 3.**
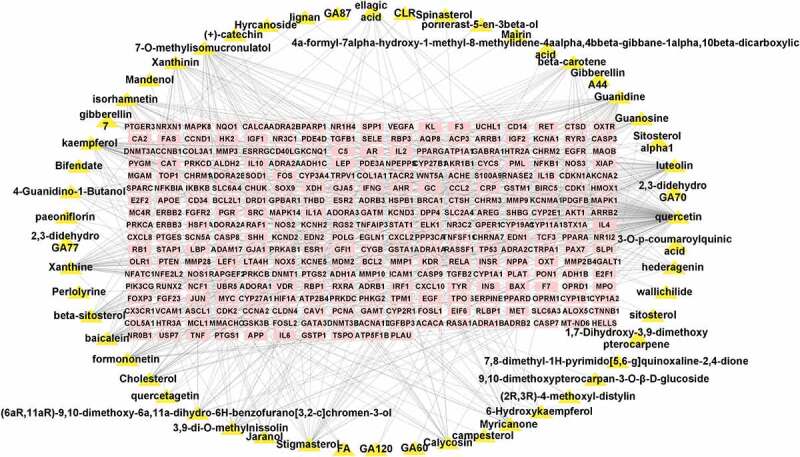
Diagram showing the relationship between the active ingredient and disease target. Pink rectangular nodes are common targets, whereas yellow triangular nodes represent the active ingredients corresponding to the targets.

A total of 3,605 active targets of MF were retrieved through the GeneCards database. An analysis of the target data of BYHWD and MF resulted in 299 common targets. The results are shown in Figure 2. The active ingredients corresponding to the common targets were retrieved from the BYHWD database. The network relationship map (Figure 3) was constructed using Cytoscape to facilitate subsequent molecular docking analysis.

### 3.3. PPI network and core network

**Figure 4.**
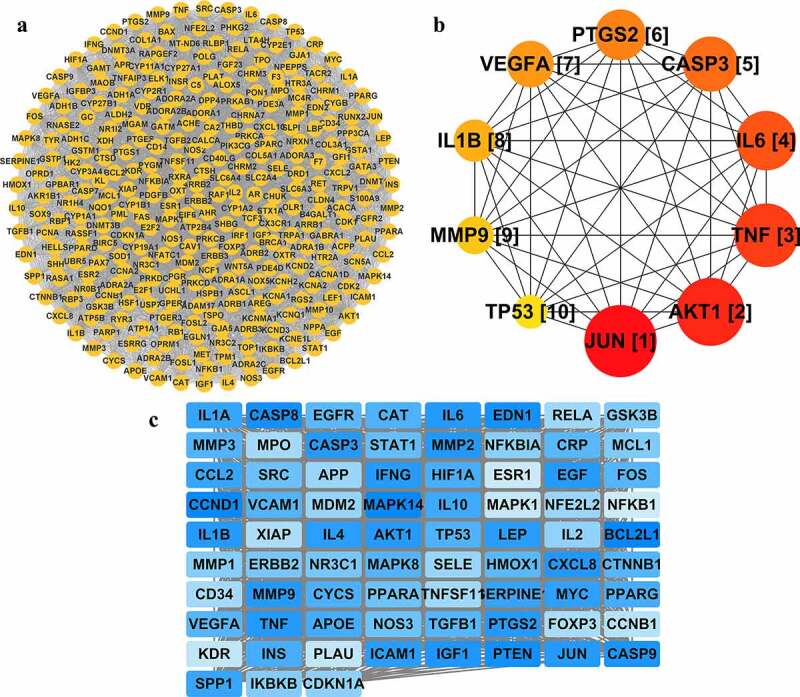
PPI network (**a**), the top 10 important targets in the PPI network (**b**), and core network (**c**). The PPI network is obtained by importing the STRING data of 299 common targets into Cytoscape. Yellow circular nodes represent common targets. Nodes are labeled with a corresponding ranking, whereas a deeper color and larger circle indicate a more important role in the network. A core network with a score of 57 involves 75 targets, where the shade of the blue rectangular node color represents the score of that node. A higher score results in a darker color, such as IL-6 and MMP9, and other dark nodes are critical targets in the network.

An analysis of 299 common targets imported into the STRING database showed that 295 targets were involved in the PPI network (four targets, *STAP1, MMACI, AQP8, MMP28* had almost no effect on the PPI network), involving 6,151 edges. These were imported into Cytoscape to obtain Figure 4(a). After analysis using CytoHubba and MCC algorithms, the top 10 essential targets, such as JUN and AKT1, are shown as bubble charts (Figure 4(b)), and the darker color represents a more critical influence of the target in the protein interaction relationship.

The MCODE plug-in was used to select a core network with a score of 57 (Figure 4 (c)). This core network contained 75 nodes with gradient colors, which were set according to the degree of darkness. The color coding made it possible to conclude that dark-colored nodes such as IL-6 and MMP9 were key targets for BYHWD to act effectively on MF.

### 3.4. GO and KEGG pathway enrichment analyses

**Figure 5.**
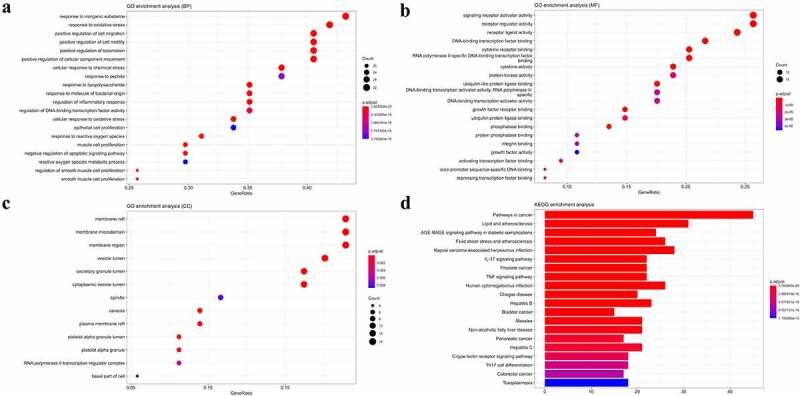
GO (**a**) (**b**) (**c**) and KEGG pathway (**d**) enrichment. The 75 core targets were subjected to enrichment analysis with a *p*-threshold set at 0.01. The top 20 entries were selected for analysis. Among the KEGG-enriched signaling pathways, the IL-17 signaling pathway was selected for the study. A smaller *p*-value indicates higher enrichment. In the bar and bubble plots, a longer bar or larger bubble indicates a more number of genes were enriched.

The 75 core targets were subjected to GO and KEGG enrichment analyses. The GO enrichment showed (Figure 5(a–c)) that genes related to biological processes were largely related to oxidative stress, lipopolysaccharides, and reactive oxygen metabolic processes. Those related to molecular functions were largely related to growth factor activity, cytokine activity, and receptor-ligand activity. Those related to cellular composition were biofilms and cell bases.

The KEGG pathway analysis showed the top 20 enriched signaling pathways (Figure 5(d)), in which the IL-17 signaling pathway with strong relevance to MF was selected for analysis.

### 3.5. Network diagrams of the relationship between signaling pathways, active ingredients, and herbs

**Figure 6.**
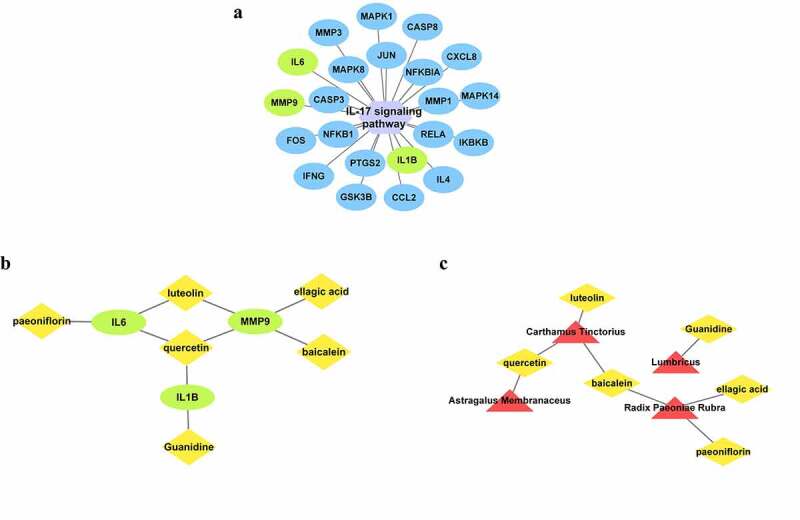
BYHWD and MF cross-target–critical signaling pathway (**a**), purple hexagonal nodes represent signaling pathways, and blue and green oval nodes represent core targets in the pathway. Critical target–active ingredient (**b**), yellow prismatic nodes represent related active ingredients, green circular nodes represent core targets being studied; and active ingredient–herbal medicine (**c**) relationship diagram. Yellow diamond nodes represent functional ingredients, whereas red triangular nodes represent herbal medicines. Based on the top 10 data in the PPI network, IL-6, MMP9, and IL-1β were selected among the 22 core targets contained in the IL-17 signaling pathway. The corresponding active ingredients and herbal medicines were collected for study.

The IL-17 signaling pathway contains 22 targets (Figure 6(a)). IL-6, MMP9, and IL-1β were selected based on the top 10 targets derived from the CytoHubba and MCC algorithms. Moreover, active molecules associated with the three essential targets were collected. The correspondence between them was imported into Cytoscape for visualization (Figure 6(b)) and data for molecular docking were constructed. In addition, a network diagram showing the relationship between the above active ingredients and herbal medicines was constructed simultaneously (Figure 6(c)), which allowed the identification of herbal medicines and herbal ingredients involved in the disease.

### 3.6. Drug-containing serum inhibits cell migration

**Figure 7.**
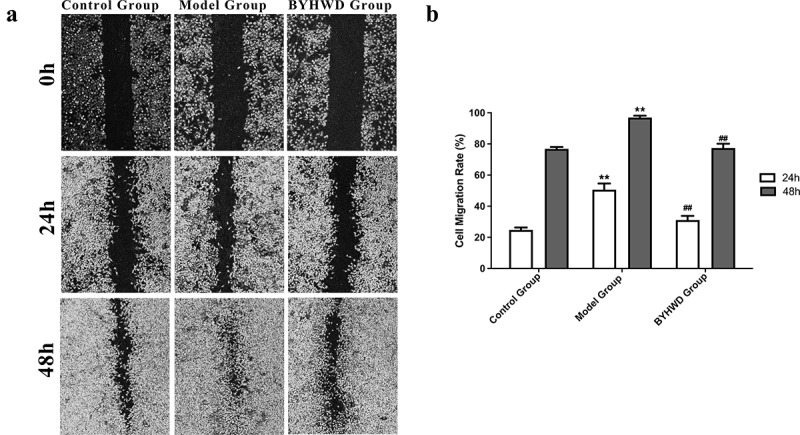
The drug-containing serum inhibits cell migration. (a) Representative microscopic fields from the migration assay. (b) Quantitative representation of RCF migration after 24 h and 48 h. *p < 0.05, **p < 0.01 vs. control group; ^#^*p* < 0.05, ^##^*p* < 0.01 vs. model group. Results are shown as mean ± SD of four individual experiments.

Results of the cell scratch assay are shown in Figure 7. The cells in the model group migrated and rapidly covered the scratch wound. The migration ability of cells in the model group was inhibited by incubating the cells with serum obtained from BYHWD-treated rats. A significant statistical difference was found between the two groups.

### 3.7. Effect on cell cycle

**Figure 8.**
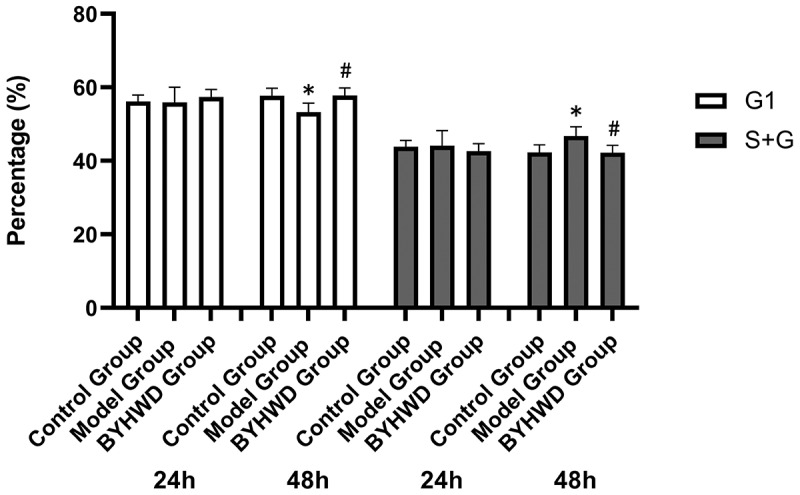
Quantitative representations of the effects of serum from the BYHWD-treated rats on cell cycle progression at 24 h and 48 h. The control group used 10% blank rat serum, whereas the model group and BYHWD group were induced with 10^−7^ mol/L ANG II for 24 h and incubated with 10% blank rat serum and 10% BYHWD-containing serum, respectively. * *p* < 0.05, **p < 0.01 vs. control group; ^#^*p* < 0.05, ^##^*p* < 0.01 vs. model group. Results are shown as mean ± SD of four individual experiments.

Figure 8 demonstrates that the number of cells in the G1 phase in the BYHWD group was higher than that in the model group after 48 h of treatment with the drug-containing serum. However, the serum of BYHWD gavage rats reduced the proportion of S + G2 cells, and the difference was significant (*p* < 0.05). This detection method showed that the administration of the drug inhibited cell division and prevented the excessive proliferation of RCFs.

### 3.8. Effect on cell microstructure

**Figure 9.**
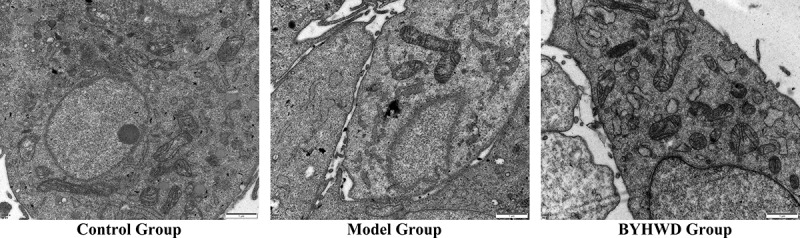
Transmission electron microscopic images of rat cardiac fibroblasts (RCFs) (magnification: 20,000×). For the control group, the nucleus was smaller and the cytoplasm had more mitochondria and endoplasmic reticulum. In the model group, a larger nucleus with fewer mitochondria and endoplasmic reticulum in the cytoplasm were observed, whereas the number of mitochondria and endoplasmic reticulum in the cytoplasm was restored in the BYHWD group.

The nucleus of cells in the model group was large, and the number of mitochondria and endoplasmic reticulum in the cytoplasm decreased. Treatment with serum from the BYHWD-treated rats restored the number of mitochondria and endoplasmic reticulum. The results are shown in Figure 9.

### 3.9. Effect of BYHWD on the mRNA expression of IL-6, IL-1β, and MMP9 in Ang II-stimulated RCFs

**Figure 10.**
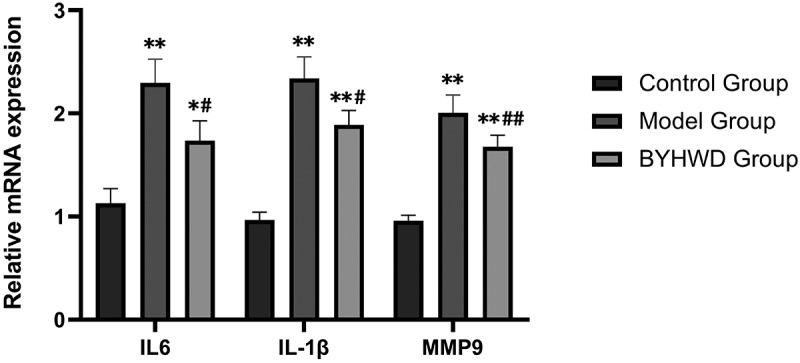
Quantitative analysis of IL-6, IL-1β, and MMP9 mRNAs. *p < 0.05, **p < 0.01 vs. control group; ^#^*p* < 0.05, ^##^*p* < 0.01 vs. model group. Results are shown as mean ± SD of three individual experiments.

Compared with the control group, we observed an increase in the mRNA content of IL-6, IL-1β, and MMP9 after Ang II induction of RCFs. However, culturing the cells with BYHWD gavage rat serum suppressed the effects of Ang II induction, and the expression of IL-6, IL-1β, and MMP9 mRNA was effectively reduced compared with the model group. The detailed statistical results are shown in Figure 10.

### 3.10. BYHWD reduces IL-6, IL-1β, and MMP9 protein content following Ang II-mediated induction of RCFs

**Figure 11.**
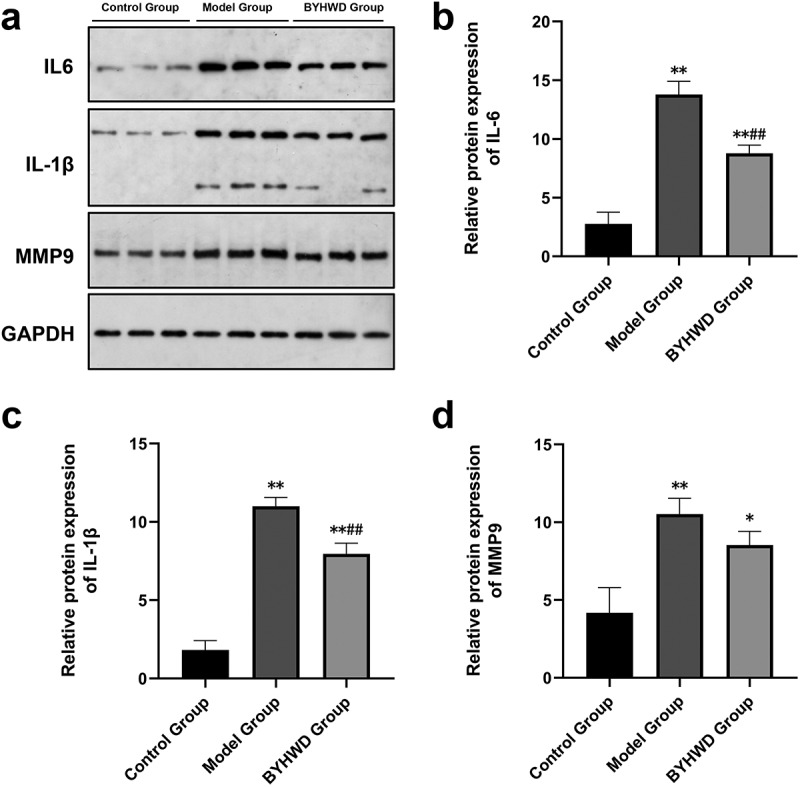
BYHWD affects the expression of pro-inflammatory factors in rat cardiac fibroblasts (RCFs). After setting up the control group, model group, and BYHWD group, the dosing treatment was performed for 48 h. Western blotting was performed with GAPDH as the internal reference. The figure shows the electrophoretic results (a), followed by optical density values to derive statistical results for the protein expression content of IL-6 (b), IL-1β (c), and MMP9 (d). *p < 0.05, **p < 0.01 vs. control group; ^#^*p* < 0.05, ^##^*p* < 0.01 vs. model group. Results are shown as mean ± SD of three individual experiments.

BYHWD reduced the expression of IL-6, IL-1β, and MMP9. As shown in Figure 11 (a–d), the protein expression of IL-6, IL-1β, and MMP9 was increased in the model group compared with that in the blank control group. A significant difference was observed between the two groups. Compared with the model group, the protein expression values were decreased in the dosing group, with a significant difference between the two groups, demonstrating the influence of BYHWD on protein expression.

### 3.11. Validation of the interaction of active ingredients with target genes

**Figure 12.**
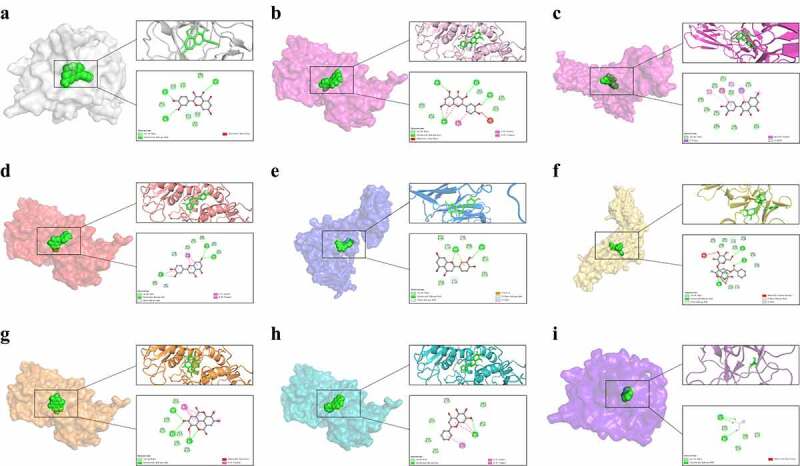
Molecular docking of critical genes to active ingredients was combined, and nine groups were visualized for analysis. These nine groups were quercetin acting on IL-1β (a), quercetin acting on MMP9 (b), quercetin acting on IL-6 (c), luteolin acting on MMP9 (d), luteolin acting on IL6 (e), paeoniflorin acting on IL-6 (f), ellagic acid acting on MMP9 (g), baicalein acting on MMP9 (h), and guanidine acting on IL-1β (i).

Among the relevant signaling pathways enriched by KEGG, we selected the top-ranked IL-17 signaling pathway containing several essential proteins involved in inflammation. Molecular docking was performed between the relevant proteins and the previously collected active ingredients; docking results are shown in Table 2. A binding affinity lower than or equal to −5 kcal/mol was considered strong. All nine groups exhibited good binding properties, except for the binding of guanidine with IL-1β, which had a binding energy higher than −5 kcal/mol. For these nine combinations, we used the visualization method as shown in Figure 12, which can be used to visualize the specific binding of different compounds to proteins.

**Table 2.** The combination of the best docking model energy

Compound	Protein	PDB ID	Affinity (kcal/mol)
quercetin	IL-1β	5R85	−7.2
quercetin	MMP9	4JIJ	−7.2
quercetin	IL6	1N26	−7.0
luteolin	MMP9	4JIJ	−7.0
luteolin	IL6	1N26	−6.8
paeoniflorin	IL6	1N26	−6.8
ellagic acid	MMP9	4JIJ	−6.7
baicalein	MMP9	4JIJ	−6.6
Guanidine	IL-1β	5R85	−3.0

## 4. Discussion

Cardiac remodeling refers to a common pathological manifestation of several cardiovascular diseases in the end stage, where cardiac remodeling is characterized as MF [27,28]. To study MF, we used BYHWD as the study object and used a combination of network pharmacology and cellular experiments to decipher the specific mechanism underlying the inhibitory activity of BYHWD against MF and its therapeutic potential for MF.

We first used the network pharmacology approach to screen the active ingredients in BYHWD using a combination of various databases. After screening and matching with differential genes of MF, we obtained 299 common targets, which were used for our further study. We constructed a PPI network and extracted the core network, 75 core targets were finally enriched for KEGG analysis. The major enriched pathways obtained were IL-17 and tumor necrosis factor (TNF) signaling pathways Figure 5(d). To select the pathways for further experiments, we compared the proteins in the two pathways with the results of core protein screening shown in Figure 4(b) and referred to the ranking of enrichment results. We finally used the IL-17 signaling pathway for subsequent experiments.

Currently, numerous studies have demonstrated the involvement of the IL-17 signaling pathway in the development of inflammatory responses [29,30]. IL-17 is widely known as a cytokine that triggers inflammatory diseases and regulates the metabolism of mRNAs of inflammatory factors through different pathways [31,32]. Therefore, the activation of the IL-17 signaling pathway significantly contributes to the mechanism of inflammation. To study the relationship between inflammation and MF, the experiments on mice have been carried out, which revealed that inflammation is involved in the development of MF. Moreover, TNF, IL-6, and IL-1β are up-regulated after the onset of inflammation, aggravating the inflammatory response and stimulating the proliferation of cardiac fibroblasts [33,34]. These results are consistent with those we eventually obtained through network pharmacology. In addition, BYHWD has been shown to exhibit good anti-inflammatory effects in previous experiments [35,36], which also reveals that we could verify the efficacy of BYHWD in inhibiting the MF development from the perspective of inflammatory reactions.

Ang II has been reported as a critical factor in developing pro-MF and inflammation. Moreover, it induces fibrosis of cardiac fibroblasts [37]. We first extracted drug-containing serum by gavage of rats with BYHWD and then cultured RCFs in vitro. Next, we stimulated the cells with Ang II to study the MF process in vitro. After simulating the disease scenario, we added the drug-containing serum and determined its therapeutic effect by measuring the cell migration capacity, cell cycle changes, and the levels of relevant inflammatory factors.

We found that the cell migration was not inhibited well in the BYHWD group, which could be ascribed to the upregulation of MMP9 content. MMP9, a matrix metalloproteinase (MMP), is involved in maintaining the balance of collagen content in the interstitial matrix. After an inflammatory response, the upregulation of MMP9 levels disrupts the balance between the production and degradation of the extracellular interstitial matrix [38], thus enhancing cell migration [39,40]. Data from the BYHWD group indicate that BYHWD can reduce the proportion of RCFs in the S + G2 phase and inhibit cell division and proliferation. During ventricular remodeling, mitochondria in cardiac myocytes are severely affected. Transmission electron microscopy revealed a reduced number of mitochondria and endoplasmic reticulum inside the cells in the model group, and the metabolism of the cells was affected.

To study inflammatory factors IL-6 and IL-1β, as the core members of the IL-17 signaling pathway [41], we measured the expression of mRNA and related proteins. We demonstrated that the continuous release of IL-6 and IL-1β following the activation of this signaling pathway resulted in left ventricular MF, collagen production, and apoptosis [42,43]. Compared with the model group, the levels of relevant inflammatory factors were down-regulated in the BYHWD group, indicating that BYHWD suppressed the inflammatory response to a certain extent.

Compared to the traditional network pharmacology approach [44,45], we added experimental validation to it to better determine the inhibitory effect of BYHWD against inflammatory response and MF. To further study the active ingredients that affect the IL-17 signaling pathway, we used the molecular docking technique to dock the potential functional components with IL-6, IL-1β, and MMP9. Molecular docking is used to study the binding between proteins and small molecule compounds through computer processing [46]. Quercetin, luteolin, paeoniflorin, ellagic acid, and baicalein showed promising docking results and could bind firmly to the target proteins. The molecular docking results can be used for further conducting subsequent drug experiments [47].

In conclusion, we used a combination of network pharmacology and cellular experiments and showed that BYHWD has great potential for treating inflammation and MF. In addition, it has high efficacy and great potential for the enrichment of multiple active ingredients. We verified the findings successfully through in vitro experiments. However, our study also had certain limitations. First, the databases involved in collecting experimental data may have affected our conclusions due to the accuracy of the information contained therein. Second, we only studied the therapeutic effects of BYHWD against MF; we did not conduct specific studies on its active ingredients. For certain active ingredients, we plan to conduct specific experiments in the future to understand the therapeutic effects of BYHWD more comprehensively.

## 5. Conclusion

In the present study, we used network pharmacology techniques to identify the potential mechanism of action of BYHWD against MF. We performed relevant cellular experiments and demonstrated that BYHWD down-regulates the expression of IL-6, IL-1β, and MMP9 in the IL-17 signaling pathway, which could reduce inflammation and inhibit MF.

## Data Availability

The data used to support the findings of this study are available from the corresponding author upon request. All the data was downloaded from here: [GeneCards] at [https://www.genecards.org/], [Traditional Chinese Medicine Systems Pharmacology Database (TCMSP)] at [http://tcmspw.com/tcmsp.php], [BATMAN-TCM] at [http://bionet.ncpsb.org/batman-tcm/], [UniProt] at [https://www.uniprot.org/], [STRING] at [https://cn.string-db.org/], [HIPLOT] at [https://hiplot.com.cn/]
